# Antibiotic-laden Arthroplasty with a Novel Design of Cement Mould and Metallic Endoskeleton for Treatment of Hip Infection, an Inexpensive Alternative: A Preliminary Report of Two Cases

**DOI:** 10.5704/MOJ.1707.015

**Published:** 2017-07

**Authors:** CCH William, S Simmrat, AM Suhaeb

**Affiliations:** Department of Orthopaedics, University Malaya, Kuala Lumpur, Malaysia

**Keywords:** antibiotic-impregnated, cement spacer, hip cement mould, metallic endoskeleton, hip arthroplasty infection

## Abstract

Infection of the hip after implant fixation is an uncommon yet devastating complication that results in poor long-term outcome. The gold standard treatment for chronic infection after hip arthroplasty is a two-stage protocol: eradication of infection, follow by re-implantation arthroplasty. The use of interim antibiotic-laden cement spacer has become a popular procedure to maintain hip joint function and provide antibiotic elution simultaneously before re-implantation. However, antibiotic cement spacer is mechanically weak and breaks if overloaded. Therefore, we designed a cement mould with metallic endoskeleton with the aim of creating a stronger, inexpensive, antibiotic-impregnated spacer resembling a unipolar arthroplasty. We report two cases of severe hip joint infection after implant fixation (bipolar hemiarthroplasty, screw fixation neck of femur). Both patients had undergone first stage surgery of debridement and articulating antibiotic cement insertion using our design. Although the second stage surgery was planned for these patients, both patients delayed the operation in view of good functional status after a year walking with the antibiotic cement spacer. These cases showed that the mechanical property of the new antibiotic cement spacer was promising but further mechanical studies upon this new endoskeleton design are required.

## Introduction

Implant infection in a hip joint is a rare yet debilitating complication, with a prevalence of 0.5% to 3%. The gold standard treatment for chronic infection of the hip joint after hip arthroplasty is a two-stage revision. First stage involves implant removal, appropriate antibiotic therapy and an interim antibiotic-laden cement hip spacer. The second stage is reimplantation surgery once infection is eradicated^1^. Antibiotic-laden articulating spacers achieve infection control by local tissue antibiotic drug elution with an added advantage of preserving limb length, joint mobility, and preventing joint contracture^2^. However, cement spacers are mechanically weak and may fracture while awaiting revision surgery^3^. The presence of an endoskeleton and standardization of antibiotic cement preparation are designed to overcome this mechanical failure of the articulating spacer^4^. Albeit various metallic endoskeletons are available in the market, it is unclear whether different shape and material used for endoskeleton affects the function, drug elution and mechanical properties of the cement spacer^5^. We present a novel design of an antibiotic cement mould with an endoskeleton to create an inexpensive antibiotic-laden cement spacer mechanically strong enough to be used in two cases with implant infection involving the hip as an interim arthroplasty replacement.

## Case Report

### Case One

**Fig. 1:**
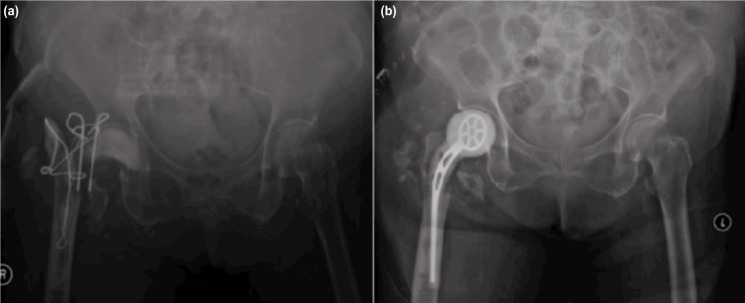
(a) Removal of right infected bipolar hemiarthroplasty, hand mould antibiotic cement was inserted into acetabulum and antibiotic beads on wire was placed in proximal femoral canal. Tension band wire over greater trochanter of femur was attempted to keep the antibiotic cement in situ and (b) A week after the initial debridement, second look surgery with insertion of antibiotic-laden right hip spacer was performed.

A 76-year old lady, with underlying diabetes mellitus and hypertension, gave a history of a fall in 2014 and had sustained fracture of the right neck of femur. She was treated surgically with bipolar hemiarthroplasty and recovered uneventfully. However, five months into rehabilitation, patient had sudden onset of debilitating right hip pain associated with fever and swelling over the previous operative scar. A diagnosis of surgical site infection extending up to the implant was made. Meticulous surgical site debridement with temporary antibiotic cement insertion was performed as first stage surgery (Fig. 1a). There was 250 cc of pus extracted from the hip joint and osteomyelitic (OM) changes over the greater trochanter and acetabulum were noted during the operation. Patient subsequently underwent removal of right hip implant and antibiotic cement spacer insertion (Fig. 1b). She was treated with appropriate intravenous antibiotic based on tissue culture for three months as in-patient. She was discharged well with wheelchair ambulation and range of motion (ROM) physiotherapy for the hip, with the plan for second stage surgery later.

At follow-up after two years, patient was ambulating with walking stick and applying full weight over the affected limb. She was able to maintain an adequate ROM of the hip: flexion 0-90 degrees, internal and external rotations of 45 degrees. With the eradication of the infection as evident by absence of symptoms and significant reducing trend of erythrocyte sedimentation rate (ESR) and C-reactive protein (CRP). Radiograph of the hip showed resolution of OM of the acetabulum. We proposed total hip replacement to the patient but she refused any intervention in view of her age and satisfaction of her current state.

### Case Two

**Fig. 2:**
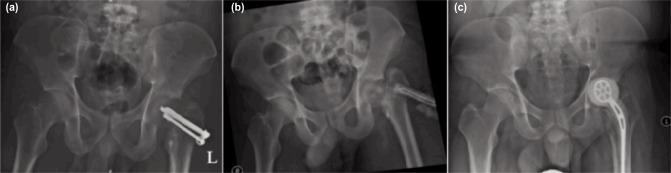
(a) On admission, radiograph showed femoral osteolysis surrounding the femoral neck screws with osteomyelitic changes of the femoral head, (b) Radiograph taken immediately after removal of implant(ROI) and insertion of antibiotic rods. Femoral neck collapsed and shorten after ROI and (c) A week after initial debridement, antibiotic-laden left hip spacer with metallic skeleton was inserted.

A 27-years old male was involved in a devastating motor vehicle accident in June 2015 and had sustained closed fracture left neck of femur, open comminuted fracture (Gustilo – grade 3B) of the midshaft of the tibia and fibula and closed fracture distal third of the right tibia. He was treated surgically with screw fixation of the left neck of femur and wound debridement with Illizarov external fixator of left tibia after the accident (Fig. 2a). The right tibia fracture was treated with cast. However, his surgical wounds broke down infested with multidrug resistant organisms. Multiple wound debridement and intravenous antibiotics were administered for six weeks before the patient was transferred to our hospital for further management. The diagnosis on admission was infected implant of left hip with septic arthritis.

CT scan and bone scan of left hip confirmed destruction of the femoral head. Hence wound debridement, removal of screws of left femoral head with temporary antibiotic cement rod insertion was performed. Intra-operatively, about 50 cc pus was found extending up to hip joint with biofilm surrounding all three screws and osteomyelitis with femoral head collapse (Fig. 2b). This was followed by debridement of the femoral head and antibiotic-loaded arthroplasty insertion after a week (Fig. 2c). Appropriate intravenous antibiotics were given for six weeks’ duration and the patient was discharged well with wheelchair ambulation, and range of motion (ROM) physiotherapy for the hip, with a plan for second stage surgery later.

On follow up after a year, patient could walk with crutches, partial weight bearing over his left lower limb. He achieved adequate ROM of left hip: flexion 0-110 degree, internal and external rotations of 60 degree. His infective markers (ESR, CRP, white cell count) were within normal range. In view of his young age, he was given the surgical option of hip fusion or replacement later in life when there was unbearable hip pain affecting the quality of life. He was content with the functionality provided by the antibiotic cement arthroplasty for now and wished to delay the operation.

### Fabrication of cement mould

**Fig. 3:**
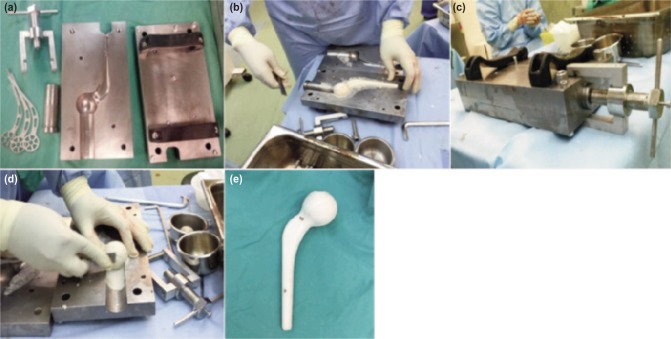
(a) Hip spacer cement fabrication set (Leonix, Pulau Pinang, Malaysia), (b) Moulding antibiotic cement in the fabrication plate, (c) Applying compression pressure in all directions on the cement mould, (d) Smoothening the sharp, irregular edges of the antibiotic cement on the hip spacer and (e) Final product of an antibiotic hip spacer with a smooth surface.

We collaborated with an implant company (Leonix, Pulau Pinang, Malaysia) to fabricate a prototype cement mould using stainless steel plates based on the Thompson unipolar hemiarthroplasty model. The newly design metallic endoskeleton was also made from stainless steel (Fig. 3a). First, 80g polymethylmethaacrylate (PMMA) bone cement (Smith & Nephew, TN, USA) was mixed, and followed by hand mix of 4g vancomycin and 4g gentamycin into PMMA with a ratio of 1:10 antibiotic: PMMA. This ratio would not alter the mechanical strength of the cement spacer and would provide maximum drug elution^5^. The cement mixture was poured on both sides of the plate and the metal endoskeleton was placed in the center (Fig. 3b). Next, both plates were compressed together and the cement pressurized with a clamp once the cement had doughy consistency. The clamp provided compression from all sides (Fig. 3c). Once the cement had hardened, all the edges were chiseled until smooth (Fig. 3d). Finally, bone file and sandpaper were used to smoothen the implant surface and washed thoroughly with normal saline to remove micro-debris. The cement spacer was then ready for fixation (Fig. 3e).

## Discussion

Periprosthetic hip infections impose a significant burden not only to the patients, but also to the healthcare system. Recently, antibiotic-laden hip spacers have become indispensable in the treatment of hip joint infections^1^. They have been successfully utilized in the treatment of proximal femur infections after osteosynthesis. Articulating antibiotic-impregnated spacers: (i) provide high dosage of antibiotic locally to the surrounding tissue by targeting the source of infection, (ii) preserve limb length and joint mobility, (iii) limit soft-tissue contracture and scarring, (iv) facilitate second-stage arthroplasty replacement^1^.

A two-stage protocol with the temporary insertion of an antibiotic-laden cement spacer was suggested as the gold standard treatment for chronic infections with a successful eradication rate of up to 95%^2^. The aim of the first stage surgery was to sterilize the infected joint after removal of the prosthesis through meticulous surgical debridement, identification of the infecting organism and antibiotic sensitivity from tissue biopsies, and administering targeted antibiotic therapy before re-implantation^2^. However, the timing of the second stage was variable but essentially depended on the resolution of infection as evident by improved hip mobility, absent clinical signs of infection, and ESR and CRP levels returning to normal values.

Despite the popular use of cement spacers, they still pose a significant risk of mechanical complications up to 50% as reported by Jung *et al*^3^. Mainly of prosthetic spacers fracture dislocation. Therefore, the logical way to improve the mechanical stability of the spacer would come from the introduction of a metal endoskeleton. Thielen *et al* showed that presence of endoskeleton doubled the strength of the spacer as compared to the spacer without. It could withstand hip joint forces up to 6.0 kN, sufficient for protected daily activities. The study also showed that all failures occurred in the upper third of the stem^4^.

Commercially, PROSTALAC is a prefabricated mould with fixed low-dose antibiotic content with different sizes available for making cement spacer. However, this device limits the size of cement spacer and dosage of antibiotic used, not to mention the high cost as well^5^. An alternative method was re-using the excised prosthetic components or femoral bone after intraoperative sterilization and adding extra support with PMMA or metallic implant such as K-wires, Rush rod, etc^5^. The disadvantage of this method was that it was time consuming and with higher risk of fractures due to cement heterogeneity and inconsistencies in mixing.

The method proposed in these two cases is cost effective as PMMA and antibiotic are the only consumables. It allows customization of the formula of bone cement and various antibiotics. The newly design metallic endoskeleton has improved the sturdiness of the articulating cement spacer evenly. Patients achieved remarkable ROM of the affected hip and functional outcome thus far. However, the prefabricated cement size is fixed and it is unclear whether different designs of endoskeleton might affect the results of interim function, infection control and mechanical complications. Currently, further mechanical studies upon this new endoskeleton design are required.

## Conflict of Interest

Fabrication of the cement mould and stainless steel endoskeleton was sponsored by Leonix, Penang, Malaysia.
